# Configural and featural processing in humans with congenital prosopagnosia.

**DOI:** 10.2478/v10053-008-0074-4

**Published:** 2010-07-01

**Authors:** Janek S. Lobmaier, Jens Bölte, Fred W. Mast, Christian Dobel

**Affiliations:** 1 School of Psychology, University of St Andrews, Scotland; 2 Department of Psychology, University of Bern, Switzerland; 3 Department of Psychology, University of Münster, Germany; 4 Institute for Biomagnetism and Biosignalanalysis, University of Münster, Germany

**Keywords:** face perception, object perception, visual cognition, prosopagnosia

## Abstract

Prosopagnosia describes the failure to recognize faces, a deficiency that can be devastating in social interactions. Cases of acquired prosopagnosia have often been described over the last century. In recent years, more and more cases of congenital prosopagnosia (CP) have been reported. In the present study we tried to determine possible cognitive characteristics of this impairment. We used scrambled and blurred images of faces, houses, and sugar bowls to separate featural processing strategies from configural processing strategies. This served to investigate whether congenital prosopagnosia results from process-specific deficiencies, or whether it is a face-specific impairment. Using a delayed matching paradigm, 6 individuals with CP and 6 matched healthy controls indicated whether an intact test stimulus was the same identity as a previously presented scrambled or blurred cue stimulus. Analyses of *d´* values indicated that congenital prosopagnosia is a face-specific deficit, but that this shortcoming is particularly pronounced for processing configural facial information.

## Introduction

Faces are a highly complex three-dimensional object type. Despite this complexity, human beings are able to discriminate between innumerable individuals with relative ease, especially if the faces are familiar. Faces are biologically relevant for human beings and it is safe to say that adults, at least, are experts in face processing. Failure in recognizing faces can have severe consequences for individuals suffering from this deficiency, a dysfunction referred to as *prosopagnosia*. Prosopagnosia has often been attributed as a consequence of brain damage in face-specific areas (Barton, Press, Keenan, & O’Connor, 2002; Bodamer, 1947). Recently, an increasing number of cases of prosopagnosia have been reported in the absence of any acquired brain lesion (Behrmann & Avidan, 2005; Carbon, Gruter, Weber, & Lueschow, 2007; Duchaine & Nakayama, 2004; Kress & Daum, 2003; Nunn, Postma, & Pearson, 2001). Such cases were termed almost synonymously as *developmental* or *congenital*. Since cases of developmental prosopagnosia have been reported in the literature which are due to early brain damage (Barton, Cherkasova, & O’Connor, 2001), and where an associated impairment such as Asperger syndrome is present (Duchaine, Nieminen-von Wendt, New, & Kulomaki, 2003), we decided to use the term *congenital prosopagnosia* instead of *developmental prosopagnosia* (Behrmann & Avidan, 2005). A further reason for this choice was the increasing evidence for a strong hereditary basis of this disorder (Gruter, Gruter, & Carbon, 2008). In fact, 2 of the participants in the present study with congenital prosopagnosia (CP) are first order relatives.

Unlike acquired prosopagnosia, the cause of congenital prosopagnosia remains unclear. Some evidence suggests possible genetic causes (de Hahn, 1999; Dobel, Bölte, Aicher, & Schweinberger, 2007; Kennerknecht et al., 2006), and some authors reported structural alterations in the inferotemporal cortex (Behrmann, Avidan, Gao, & Black, 2007) and the ventral occipito-temporal cortex (Thomas et al., 2009). The question as to which cognitive characteristics best describe this deficit is still under debate. In the present study, we suggested that a deficit in configural processing might characterize congenital prosopagnosia, since configural information has been demonstrated to play an important role in face processing.

A face contains complex information and different ways of processing this information have been discussed. Many authors have suggested that faces are processed holistically and are stored as a whole (Farah, Tanaka, & Drain, 1995; Farah, Wilson, Drain, & Tanaka, 1998; Leder & Carbon, 2005). Various interpretations of holistic face processing have been suggested. The pure holistic view of face recognition claims that faces are represented as whole templates without facial parts being stored explicitly (Tanaka & Farah, 1993). Other authors have suggested a differentiation between *configural* and *featural information* (Bartlett, Searcy, & Abdi, 2003; Cabeza & Kato, 2000; Farah et al., 1998; Schwaninger, Lobmaier, & Collishaw, 2002; Tanaka & Farah, 1993). *Featural information* refers to the constituent parts of a face (i.e., eyes, nose, and mouth) whereas *configural information* is understood as the spatial relationship between the constituent elements of a face (Diamond & Carey, 1986; Maurer, Le Grand, & Mondloch, 2002; Schwaninger et al., 2002). While every visual stimulus contains configural and featural information, it has been suggested that non-face objects are predominately processed in a part-based fashion (Biederman, 1987; Marr, 1982). Configural processing in turn seems to be a hallmark of face perception (Cabeza & Kato, 2000; Diamond & Carey, 1986; Farah et al., 1998; Schwaninger et al., 2002; Tanaka & Farah, 1993). It has been suggested that this dominant role of configural information in face processing results from the expertise that humans develop for faces (Diamond & Carey, 1986; Mondloch, Geldart, Maurer, & Le Grand, 2003).

A possible cognitive cause of the symptoms of congenital prosopagnosia may therefore lie in specific deficits in the processing of configural information. This assumption is supported by findings investigating the face inversion effect (FIE). The FIE describes the phenomenon whereby faces are disproportionately more difficult to recognize when viewed upside down. This decrease in performance is commonly interpreted as a result of disrupted configural processing (Leder & Bruce, 2000; Leder & Carbon, 2006). Compared to normal individuals, individuals with congenital prosopagnosia show a less pronounced FIE (Behrmann, Avidan, Marotta, & Kimchi, 2005; Duchaine, Dingle, Butterworth, & Nakayama, 2004). One study found that some individuals with prosopagnosia even show an advantage in processing inverted faces, whereas other individuals show only similar, but not advantageous, processing of inverted faces (de Gelder & Rouw, 2000). Furthermore, individuals suffering from this impairment display a more dispersed fixation pattern when recognizing faces (Schwarzer et al., 2007), which is compatible with an impairment of configural encoding. It has to be noted, however, that some studies did not suggest deficient configural processing (Duchaine, 2000). In a single case study, Duchaine reported patient B.C. who showed impairment in some aspects of face processing, but scored above average in three tests of configural processing. The author concluded from this that prosopagnosia may exist without configural processing deficits.

From the findings reported above, people suffering from CP may rely more strongly on featural information and, as a consequence, process faces like objects. The aim of the present study is to examine whether congenital prosopagnosia is due to a general difficulty with configural processing, or whether it is a face-specific impairment. In other words, we examined whether hindered face recognition in people with CP may be characterized by an inability to adequately process configural information. Alternatively, people with CP may have a highly face-specific impairment, in which case a weaker performance could be expected in configural and featural face processing alike, but not in processing configural or featural aspects of other objects. We addressed this question by comparing the configural and featural processing of faces, houses, and sugar bowls. Following Schwaninger et al. (2002) and Lobmaier & Mast (2007), we defined *featural information* as the local information contained in the individual parts. *Configural information* is understood as the spatial interrelationships between the parts. Similar to faces, houses are rather complex stimuli that are made up of distinct features displayed in a specific configuration. Sugar bowls are less complex. They are made up of a smaller number of features (body, lid, handles), but, as with faces and houses, the configuration of these features is predetermined. Comparing three different object types permits a better determination of the stimulus specificity of congenital prosopagnosia. We separately investigated configural and featural processing using scrambled and blurred versions of the three types of stimuli (Collishaw & Hole, 2000; Lobmaier & Mast, 2007; Schwaninger et al., 2002). Segregating the constituent parts of a stimulus and rearranging these disrupts configural information while preserving detailed featural information. Applying a sufficient blur to a stimulus preserves the configuration, but impairs detailed featural information. Previous studies investigating configural and featural (face) processing often used stimuli where the configuration was changed (Haig, 1984; Macho & Leder, 1998; Searcy & Bartlett, 1996; Tanaka & Sengco, 1997) or where the features were changed (Farah et al., 1998; Searcy & Bartlett, 1996; Sergent, 1984; Tanaka & Farah, 1993). Changing one kind of information is problematic, as configural changes may also involve featural changes and vice versa (Rakover, 2002). For example, stretching the inter-eye distance may result in the bridge of the nose appearing wider. Although the use of scrambled and blurred stimuli is yet another method of teasing apart configural and featural processing, it might be advantageous over other methods as it enables the examination of one processing strategy without tampering with the other.

We used a delayed same-different task to determine whether the perceptual weakness found in individuals with CP is domain specific (i.e., restricted to the recognition of faces), or process specific (i.e., restricted to configural processing). A cue image was presented which was either blurred or scrambled, followed by an intact test stimulus. Participants judged whether the test and cue stimuli were the same. Critically, the test stimuli which had to be matched to the blurred and scrambled cue stimuli were intact. The logic behind this is that matching intact faces, houses and sugar bowls to the cue stimulus activates featural processing strategies in the case of scrambled cues and configural processing strategies when the cue was blurred. Moreover, since the test stimuli were always intact in both conditions, any behavioural differences resulting from visual differences in the test face can be excluded. The participants had to use the information that was available to them (either configural or featural information) to solve the task. This constitutes a significant advantage over using inverted or spatially and featurally manipulated stimuli.

We compared the matching performance of intact to scrambled and intact to blurred versions of faces, houses, and sugar bowls to test the following predictions: If congenital prosopagnosia is a result of process-specific difficulties, individuals with CP should perform worse in blurred compared to scrambled trials of all stimulus types. In the blurred trials, participants of the control group should outperform individuals with CP; in the scrambled conditions, performance should be comparable in both groups. In contrast, if congenital prosopagnosia is a domain-specific dysfunction, people with congenital prosopagnosia should have more difficulty in matching faces than participants in the control group in both scrambled and blurred conditions. However, no difference between the two groups would be expected for houses and sugar bowls. Finally, CP could be the result of a process-specific deficit that is especially pronounced in face processing. In this case we would expect configural processing to be impaired only in faces, while both configural and featural processing of other object classes would be unimpaired.

## Method

### Participants

Six individuals with CP took part in this study; G.H. (56 years old, female), M.H. (27 years old, male) and X.G. (54 years old, male) have been described in detail in two other studies (Dobel et al., 2007; Dobel, Putsche, Zwitserlood, & Junghofer, 2008), whilst B.T. (28 years old, female), L.O. (22 years old, female) were also described in an earlier study (Dobel et al., 2008). These participants also took part in a study on biological motion (Lange et al., 2009), and each of them showed an impaired performance compared to controls in at least one test of biological motion.

Subject S.G. (22 years old, female, 13 years of education; profession: nurse) had not participated in earlier studies. Like the other participants, she was examined with the test battery as described in detail in Dobel et al. (2007) . All individuals with CP, including S.G., were characterized by a failure to recognize famous people (the Bielefelder Famous Faces Test; Fast, Fujiwara, & Markowitsch, in press) and by very delayed responses in delayed matching to sample tasks when comparing faces to eye glasses. All individuals with CP were also tested with the Benton face recognition task (Benton, Sivan, Hamsher, Varney, & Spreen, 1983). At first glance it might be surprising that the individuals with CP passed the Benton face recognition test with remarkably good results. However, we were not the first to find such a result. Duchaine and Nakayama (2004) observed a normal performance of 7 of their 11 congenital prosopagnosic subjects. On top of this, Duchaine and Weidenfeld (2002) raised serious doubts that normal scores on the Benton test demonstrated unimpaired face processing. The performance of their participants in neuropsychological test batteries for more general visual abilities, such as the Visual Object and Space Perception Battery (VOSP, Warrington & James, 1992), was inconspicuous in almost all of them. In two subtests of the VOSP (progressive silhouettes, position discrimination), B.T. performed below the critical cut-off level. Subject L.O. performed at the cut-off level in the screening-test and progressive silhouettes of the VOSP. Subject S.G. always scored above the cut-off level. Thus, based on these tests, we found no striking evidence for any neuropsychological deficits aside from the impairment in face processing (see Table 1; for a short description of all tests, see Appendix A).

**Table 1.** Test Scores and Results from Neuropsychological Test Batteries and Other Experiments.

	Controls	G.H.	M.H.	X.G.	L.O.	B.T.	S.G.
	Visual Object and Space Perception Battery						
Screening (*ns*)	20 ± 0,0	18	20	19	15	18	20
Incomplete Letters(*ns*)	20 ± 0,0	20	20	20	20	20	19
Silhouettes (*ns*)	26 ± 4,7	27	29	22	16	16	21
Object Decision (*ns*)	18 ± 0,5	20	18	18	18	18	18
Progressive Silhouettes (*ns*)	8 ± 3,1	^b^	4	10	9	13	10
Dot count (*ns*)	10 ± 0,0	10	10	10	10	10	10
Position Discrimination (*ns*)	20 ± 0,4	19	20	20	20	16	20
Number Location (*ns*)	10 ± 0,8	10	9	10	10	10	10
Cube Analysis (*ns*)	10 ± 0,0	10	10	10	10	10	10
	Snodgrass Picture						
Naming (% correct) (*ns*)	100 ± 0,0	97	100	97	100	100	100
	Bielefelder Famous Faces Test						
% recognized faces from visual cue (**)	73 ± 12,3	30	31	47	3	40	31
	Delayed Matching to Sample of faces and glasses
^a^Latencies: glasses (*ns*)	1,4 ± .4	1,5	2,6	2,3	1,0	1,7	1,2
^a^Latencies: faces (*)	1,9 ± .5	2,8	4,0	4,1	3,2	4,2	3,9
% correct: glasses (*ns*)	95 ± 6,3	95	100	90	95	95	95
% correct: faces (*)	86 ± 7,5	95	100	90	90	85	90
Benton Facial Recognition Test (*ns*)	48 ± 2,1	48	48	43	49	39	48
Judgment of (% correct):							
Emotional expression (*ns*)	99 ± 2,7	80	93	87	93	100	87
Gender (*ns*)	100 ± 0,0	100	100	100	100	100	100
Age (*ns*)	100 ± 0,0	100	100	100	100	100	100
Gaze direction (*ns*)	100 ± 0,0	100	100	100	100	100	100

*Note*. Data from controls (*N*= 6) as well as from G.H., M.H. and X.G. are taken from Dobel, Bolte, Aicher, and Schweinberger (2007). Indicated next to the test is whether there was a statistically significant difference between groups (*ns* = no difference, *p <.05, **p < .01).* Indicates significant at the .005 level.

a Latencies – in seconds.

b Missing value for G.H. in progressive silhouettes: G.H. was tested by a different group on an earlier occasion with the progressive silhouettes and remembered the two items, so we therefore could not retest her on the progressive silhouettes.

The control group consisted of 6 healthy controls: H.J. (58 years old, female), L.E. (30 years old, male), A.L. (58 years old, male), A.S. (29 years old, female), L.G. (23 years old, female) and S.H. (25 years old, female). All were personal acquaintances (friends or family members) of the experimenters and none showed any signs of face perception impairment. All participants were naive regarding the purpose of the experiment. All reported normal colour vision as this was previously tested in medical examinations for the army, to receive driving licences or to determine whether glasses were needed. All participants reported normal or corrected to normal vision and were treated according to the declaration of Helsinki.

### Apparatus

The study was conducted using a laptop computer running on Windows NT, using Superlab Pro 2.0.2 software. The stimuli were presented on a 15” flat screen. The participants were seated on a height-adjustable chair and responded by pressing the “f” and “j” keys on the keyboard.

### Stimuli

**Figure 1.**
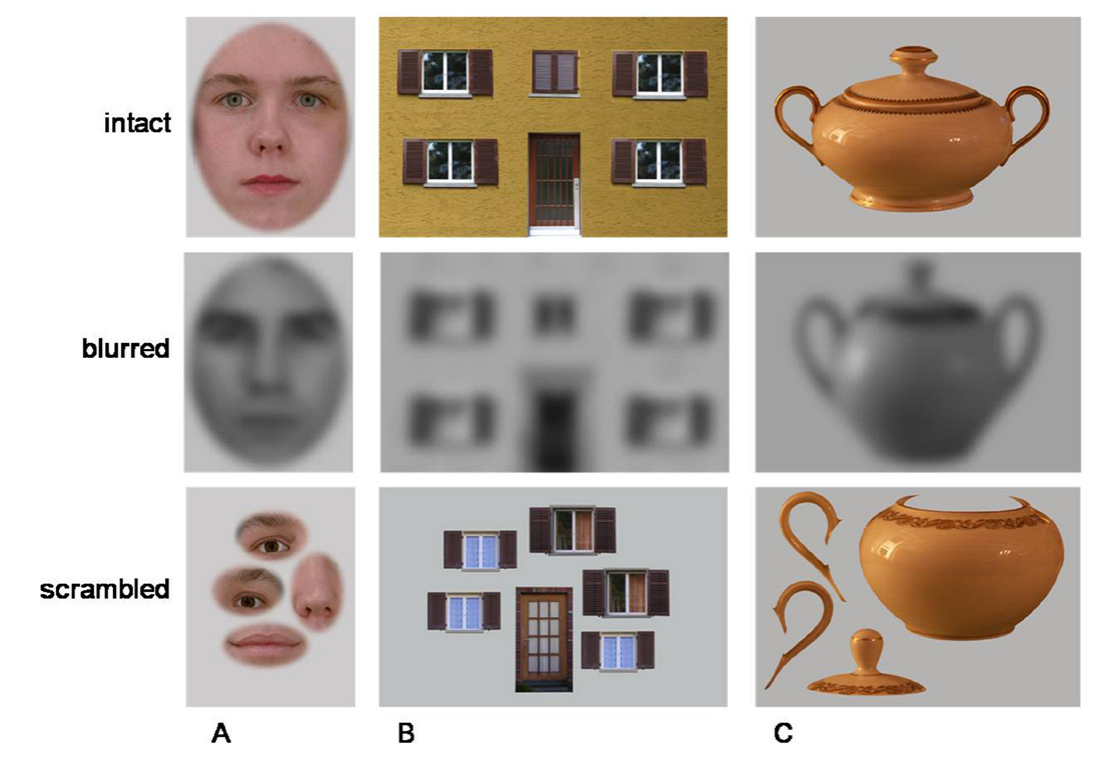
Examples of stimuli used: Intact, blurred, and scrambled versions of face (A), house (B), sugar bowl stimuli (C). Intact and scrambled stimuli were presented in colour.

Intact, scrambled and blurred versions of 40 faces, houses, and sugar bowls were used as stimuli. The face stimuli were similar to those described in a previous study (Lobmaier & Mast, 2007). The outer features of the faces, such as head shape and hair line, were discarded by cutting out the faces using the Elliptical Marquee Tool (320 × 410 pixels) provided by Adobe Photoshop 7.0. Thus, all the faces were the same in size and shape (110 × 140 mm). Blurred faces were created from the intact faces in two steps. Firstly, colour information was discarded from the intact faces because colour does not contain any space-related information. We therefore defined colour as featural information (Lobmaier & Mast, 2007, 2008; Schwaninger et al., 2002). Secondly, the faces were blurred using a Gaussian filter^1^ provided by Photoshop 7.0. Scrambled faces were obtained by cutting out the eyes, mouth, and nose using the elliptical tool described above and by re-locating these in non-natural positions. Colour information was retained in the scrambled stimuli, since according to our definition of featural information, colour is important local information. The house stimuli were composites of six features (five windows, one door) placed on individual facades. No feature was used for more than one house and the features were arranged in a natural position, thus creating a distinct individual configuration. All the houses shared the standard outer shape (230 × 140 mm). Blurred versions were created with the Gaussian filter provided by Photoshop 7.0, using a radius of 10 pixels, which resulted in a blur level comparable to the one used for faces. Scrambled houses were created by placing the features onto a grey background in non-natural positions. The sugar bowls were all of similar, but not identical, shape. Blurred sugar bowls were created using the same procedure as for the blurred houses; scrambled versions were created by cutting out handles, lid and the body and placing them onto a grey background in non-natural positions. All object types were scrambled in different versions which were presented randomly, thus preventing the participants from anticipating the exact location of the features. Examples of the stimuli can be seen in Figure 1.

### Task and procedure

In a sequential matching task participants were required to match a blurred or a scrambled cue stimulus with a subsequent intact test stimulus. Each trial consisted of a fixation cross which appeared for 500 ms, followed by a blurred or scrambled cue (face/house/sugar bowl). After 2000 ms the cue disappeared and a random dot mask appeared. This mask was included to avoid afterimages and thus to minimize the possible use of a picture-matching strategy. The mask was replaced after 1000 ms by an intact target (face/house/sugar bowl). This target disappeared after 5000 ms, or as soon as an answer key was pressed. The task was to decide as quickly and accurately as possible whether the cue and the target belonged to the same individual face, house or sugar bowl, respectively. Half of the trials were the same, the other half were different trials. The participants could go on to the next trial by pressing the space bar.

All participants gave informed consent prior to the experiment. They received written and oral instructions. The experiment consisted of two blocks of each stimulus category, each block encompassing 20 blurred and 20 scrambled trials. Different stimulus pairs were used in each block and the order of the blocks was counterbalanced across participants. Each block was approximately 10 min long. After each block participants could take a break if needed.

## Results

**Figure 2.**
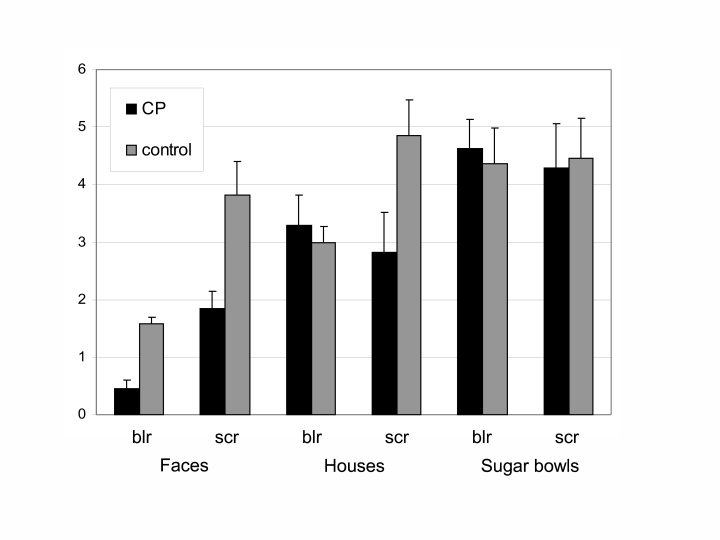
Mean *d*' values for scrambled and blurred trials, separated by group and stimulus type. Error bars depict standard errors of the mean (*SEM*). blr = blurred, scr = scrambled.

Analyses of reaction times (RT) revealed no difference between the CP and the control group (*p* = .411) and no interaction with the factor Group reached statistical significance (all *p*-values > .11), so we refrained from reporting RT data (see Appendix B for RT data). Instead, we analysed recognition accuracy in terms of *d*-prime values (*d*’). *D*-prime values were calculated for each participant in each condition by subtracting the z-transformed false alarm rates from the *z*-transformed hit rates. To get an impression of general recognition performance, we first compared the *d*’ values of all six variables against chance (*d*’ = 0). The control group recognized all faces and objects in both manipulation conditions very accurately (all *p*-values > 6.4; all *p*-values < .001). Even though the individuals with congenital prosopagnosia also performed above chance, the performances were worse than in the control group (all *p*-values > 3.13; all *p*-values < .03). The lowest *d*’ value was attained for blurred faces (see Figure 2 and Table 2).

**Table 2.** *d*' for Each Subject, Group Means and Standard Errors Separated by Stimulus Types and Type of Manipulation.

	Faces		Houses		Sugar bowls	
	Blurred	Scrambled	Blurred	Scrambled	Blurred	Scrambled
Participant	Control group					
H.J.	1,42	2,68	2,49	2,17	2,68	2,93
A.S.	1,64	3,84	2,56	4,64	6,00	6,00
L.G.	1,88	6,00	3,29	6,00	6,00	6,00
S.H.	1,68	4,64	4,04	6,00	4,64	6,00
A.L.	1,77	2,12	3,29	4,28	4,28	3,29
L.E.	1,06	3,67	2,32	6,00	2,56	2,56
^a^Group means	1,58	3,83	2,99	4,85	4,36	4,46
*SE*	0,127	0,52	0,29	0,33	0,52	0,51
Participant	Experimental group (congenital prosopagnosics)					
G.H.	0,67	2,32	4,28	4,64	4,64	2,93
B.T.	0,65	1,88	2,32	2,32	2,93	6,00
L.O.	0,13	1,90	3,67	2,17	6,00	6,00
S.G.	0,73	1,16	1,20	0,27	3,52	2,68
X.G.	-0,11	0,91	4,28	2,93	4,64	2,12
M.H.	0,65	2,93	4,04	4,64	6,00	6,00
^a^Group means	0,45	1,85	3,30	2,83	4,62	4,29
*SE*	0,14	0,30	0,51	0,68	0,51	0,77

a All group means were above chance level.

Inspection of the range of each variable in both groups revealed no overlap in performance between the groups for blurred faces (maximum CP: 0.7; minimum control: 1.1). In fact, the CP participant with the best performance was more than two standard deviations below the mean of the control group. For scrambled faces, there was a slight overlap between the groups (maximum CP: 2.9; minimum control: 2.1): 2 participants from the CP group performed within the range of the control group. For all other variables, the overlap between the groups was much larger (see Table 2). In fact, the sugar bowl performance was near ceiling for both groups, therefore the sugar bowls reduced overall variance^2^. Thus we omitted the sugar bowls from further analyses. Instead, we performed an ANOVA test including the within-participant factors object type (faces, houses) and manipulation (scrambled, blurred) and the between-participant factor group (control, CP).

The main effect of object type, *F*(2, 20) = 26.7, *MSE* = 1.442, *p* < .001, η_p_^2^= .728, revealed that the performance of both groups for face stimuli (*M* = 1.93, *SE* = 0.183) was weaker than for houses (*M* = 3.49, *SE* = 0.345). Blurred stimuli (*M* = 2.08, *SE* = 0.147) were not recognized as well as scrambled stimuli; *M* =3.34, *SE* = 0.357; *F*(1, 10) = 19.1, *MSE* = 0.993, *p* = .001, η_p_^2^= .656.

Individuals suffering from congenital prosopagnosia showed an overall weaker performance than individuals in the control group (CP: *M* = 2.11, *SE* = 0.328; control: *M* = 3.31, *SE* = 0.328), resulting in a significant group effect, *F*(1, 10) = 6.7, *MSE* = 2.584, *p* = .027, η_p_^2^ = .403.

Significant interactions further qualified the main effects. The group by manipulation interaction was significant, *F*(1, 10) = 7.6, *MSE* = 0.993, *p* = .020, η_p_^2^= .433. This interaction resulted from the fact that the performance of the control group depended on the manipulation (blurred: *M* = 2.287, *SE* = 0.208; scrambled: *M* = 4.338, *SE* = 0.505) while this was not so much the case for the individuals with prosopagnosia (blurred: *M* = 1.876, *SE* = 0.208; scrambled: *M* = 2.339, *SE* = 0.505). The object type by manipulation interaction was also significant, *F*(1, 10) = 16.4, *MSE* = 0.234, *p* = .002, η_p_^2^ = .621. Blurred faces (*M* = 1.02, *SE* = 0.094) were not discriminated as well as scrambled faces (*M* = 2.84, *SE* = 0.321), however, no such difference was evident for houses (blurred: *M* = 3.15, *SE* = 0.291; scrambled: *M* = 3.84, *SE* = 0.460). The interaction object type by group; *F*(1, 10) = 1.3, *MSE* = 1.083, *p* = .277, η_p_^2^ = .117, failed to reach significance. The three-way interaction of Object type × Manipulation × Group reached statistical significance, *F*(1, 10) = 6.9, *MSE* = 0.234, *p* = .026, η_p_^2^= .407. Post hoc analyses revealed that this three-way interaction resulted from the fact that while CPs performed equally well with scrambled and blurred houses (*p* = .262), the controls performed better with scrambled compared to blurred houses (*p* < .05). For faces, both CPs and controls performed better in the scrambled condition (both *p*-values < .01).

## Discussion

In the present study, we investigated whether congenital prosopagnosia is a face-specific dysfunction, or whether it is a result of impaired configural processing, by comparing featural and configural processing of faces, houses and sugar bowls. Because the sugar bowls were recognized near the upper performance limit and thus reduced the overall variance, we omitted them from further analyses.

The houses were generally matched more accurately than faces, and overall, scrambling a cue stimulus affected performance to a lesser degree than blurring. Participants of the control group generally outperformed the individuals with congenital prosopagnosia, which was revealed by the significant group effect. More interesting findings were revealed in the significant three-way interaction. Both groups were better at matching scrambled than blurred faces, but with the house stimuli the individuals with CP performed equally well when they were scrambled or blurred. In contrast, the control participants showed a weaker performance for blurred than for scrambled houses. The fact that individuals with CP performed equally well in the featural and configural house conditions suggests that congenital prosopagnosia is not a general impairment of configural processing. If so, we could expect a weaker performance in all blurred conditions compared to the scrambled conditions. In contrast, for face stimuli, both groups showed a weaker performance in the blurred than in the scrambled condition, while individuals with CP showed an overall weaker performance than individuals in the control group. The three-way interaction thus suggests that CPs show selective impairment in processing faces, but not when processing houses. Interestingly, we found no effects for response latencies. There was a large variance of response latencies between participants with CP, which in combination with the relatively small number of participants might have lead to the non-significant effects for latencies.

Descriptive statistics of the individual data further evaluate the findings of the analyses of variance. While the performance of the two groups overlapped for scrambled faces, there was no such overlap for blurred faces: In the blurred condition, the two groups were clearly distinct. This suggests that the deficiency of the CPs studied in the present paper is particularly pronounced in processing facial configurations.

The finding that faces were generally recognized more accurately in the scrambled condition may seem surprising, as this contrasts with other studies reporting better performance for configural than for featural face processing (Cabeza & Kato, 2000; Diamond & Carey, 1986; Farah et al., 1998; Schwaninger et al., 2002; Tanaka & Farah, 1993). However, in a previous study which also employed a scrambled-blurred paradigm, it was found that an advantage for configural processing could only be demonstrated for familiar faces (Lobmaier & Mast, 2007), whilst an advantage for featural processes was found for novel faces (see also Bombari, Mast, & Lobmaier, 2009). In the present study the stimulus faces were all unfamiliar to the participants. Had we used familiar faces or highly learned faces, we would indeed have expected an advantage in the blurred condition.

Taken together, the findings of the present study indicate that congenital prosopagnosia is in effect a face-specific impairment. Descriptive analyses of the data suggest that in CP configural face processing strategies might be particularly affected. While individuals not affected by CP allocate both configural and featural strategies when processing faces and objects, individuals with CP may fail to allocate configural strategies when processing faces. However, when CPs process other objects, such as houses, configural processing strategies do not seem to be defective. It has to be noted that the sample size of our groups was rather small for statistical group analyses, given that CP is a heterogeneous deficit (Dobel et al. 2007). Increasing the number of control participants would have resulted in more power, but an unequal number of participants would also call for a more liberal test if the smaller sample (prosopagnosics in our case) exhibited more variance than the larger sample. To avoid a liberal statistical test we matched the participants and used groups of equal size.

Other studies also attempted to explore the role of configural processing in CP, some finding evidence that CP might involve some deficits in configural processing (Behrmann et al., 2005; Duchaine et al., 2004), while others suggest that CP can occur without specific deficits in configural processing (Duchaine, 2000). The present study complements and extends previous findings since it employed a paradigm that directly differentiated between configural and featural processing strategies without altering the visual properties of the critical visual stimulus.

We note that in the present study colour information was discarded in the blurred stimuli but not in the scrambled or intact stimuli. This was due to our definition of featural and configural information, according to which configural information was restricted to spatial relations between the parts. We cannot completely rule out the possibility that the drop in performance of people with CP was purely based on configural processing deficits or whether it was a result of the lacking colour information. The fact that we found a drop in performance in both groups suggests that the lack of colour information may indeed make recognition of the blurred trials more difficult, but we see no reason why the unavailability of colour information should have a stronger impact on people with CP.

Individuals with congenital prosopagnosia often claim to rely on featural rather than configural strategies when recognizing faces (Duchaine & Nakayama, 2005). Despite the accustomed use of featural processing, CPs did not outperform the control group in any of the scrambled conditions. Although CPs performed better with scrambled than with blurred faces, this was also the case in the individuals without CP. Hence, we found no evidence suggesting that CPs use featural information more successfully than individuals with unimpaired face recognition. Rather, we suggest that, at least in our sample, individuals with CP had not acquired a featural strategy which would help them to process faces. This, however, does not deny the use of other (external) features, such as gait, voice or hairstyle.

There is increasing evidence that congenital prosopagnosia is accompanied by a reduction of left hemispheric activity in response to faces. As Bentin and colleagues (2007) pointed out, the data from Avidan and co-authors (Avidan, Hasson, Malach, & Behrmann, 2005) seemed to show that right hemispheric functioning was unimpaired in congenital prosopagnosics. In contrast, a clear reduction of activity in response to faces was visible over left hemispheric areas, specifically in the area of the fusiform gyrus. Similarly, Bentin et al. (1999) found in an ERP study that the largest difference between a congenital prosopagnosic and the controls seemed to arise over the left hemisphere. In line with these findings, an MEG study by Dobel and colleagues (2008) revealed that, compared to the controls, individuals with CP showed a reduced M170, especially over the left hemisphere. Reduced activity over the left hemisphere is slightly inconsistent with our finding that people with CP have a specific shortcoming in processing configural face information. It is usually assumed that the right hemisphere is more specialized for configural or holistic processing while the left hemisphere seems to be more responsible for analytical or featural processing (Lobmaier, Klaver, Loenneker, Martin, & Mast, 2008; Rossion et al., 2000). Our present findings suggest that although deficits in congenital prosopagnosia seem to be most pronounced for face processing, this shortcoming depends on the complex interplay of featural and configural processing strategies. While no impairment of configural processing compared to featural processing was noticeable for the house stimuli, individuals with CP showed a distinct deficit in processing configural face information. Impaired processing of faces in the face of inconspicuous processing of houses has also been reported by Duchaine and Nakayama (2006) , further underlining the evidence that faces and houses are processed by dissociated neural networks (see also Gruter, Gruter, Bell, & Carbon, 2009, Experiment 2).

Although therapies aiming to improve configural processing have been successful (Degutis, Bentin, Robertson, & D’Esposito, 2007), our results suggest that one processing strategy is unlikely to compensate for the lack of the other. This has implications on tools used to diagnose congenital prosopagnosia. Specifically, our data suggest that it is potentially misleading to simply contrast, in a dichotomous way, the recognition performance of faces to that of all other visual objects. Rather, the present findings suggest that although congenital prosopagnosia primarily affects face perception, this impairment depends on the specific allocation of configural and featural processes.

## Footnotes

1 The Gaussian filter used a sigma of 0.025 of image width in frequency space, using the equation *exp*[-*f*^2^/(2 x sigma^2^)].

2 This was confirmed in a three-way analyses of variance (ANOVA) including the factors object type (faces, houses, sugar bowls), manipulation (scrambled, blurred), and group (control, CP): There was a main effect of object type, *F*(2, 20) = 26.7, *MSE* = 1.442, *p* < 0001, η2 = .728; and of manipulation, *F*(1, 10) = 9.3. *MSE* = 1.238, *p* = .012, η_p_^2^ = .482. There was no group effect, *F*(1, 10) = 2.3, *MSE* = 0.813, *p* = .161, η_p_^2^ = .186. The following interactions were significant: between group and manipulation, *F*(1, 10) = 5.3, *MSE* = 1.238, *p* = .045, η_p_^2^ = .345; and between object and manipulation, *F*(2, 20) = 10.7, *MSE* = 0.533, *p* = .001, η_p_^2^ = .516 . The interaction object type by group, *F*(2, 20) = 2.7, *MSE* = 1.442, *p* = .095, η_p_^2^ = .210; and the three-way interaction, *F*(2, 20) = 2.8, *MSE* = 0.533, *p* = .088, η_p_^2^ = 0. 216; failed to reach significance.

## Appendix A.

### Brief description of neuropsychological tests and experiments which were employed for the diagnosis of the impairment

#### VOSP

The Visual Object and Space Perception Battery consists of a screening test and several subtests.

In the screening test, participants are presented black-and-white random patterns upon which they have to detect the presence of an "X". Incomplete letters subtest consists of letters that are partially overlaid by a random pattern. Participants have to identify the corresponding letter. In silhouettes task, silhouettes of objects were rotated around their vertical axis and have to be identified. Object decision subtest requires the identification of existing objects from non-exisiting objects. In Progressive silhouettes task, two silhouettes of objects are presented repeatedly while decreasing the recognition difficulty. Difficulty is manipulated by starting at an unusual point of view to the regular lateral view. In the dot count task participants have to identify the number of randomly located dots ranging between 5 and 9. Position discrimination task requires the identification of one of two test displays in which a dot is located right in the centre. For the number location subtest participants have to compare two displays, one containing randomly arranged numbers, the other a dot located at a specific position. Participants have to indicate which number is located at the location of the dot. In cube analysis task, participants are presented 2D versions of stacked cubes in 3D. The task is to count the number of cubes including the ones that are not visible in the 2D display.

#### Snodgrarass Picture Naming Task

In this task participants had to name black-and-white drawings of objects from the categories fruit, tools, animals, and clothing.

#### Bielefelder Famous Faces Test

For the presented score of the Bielefelder Famous Faces Test, participants had to identify famous persons by name or biographical information.

The delayed matching to sample task required the identification of a target stimulus from a distractor after a delay of 3.5 s following the encoding phase of the target. Stimuli consisting of faces or eye-glasses were presented in different views during the encoding and the identification phase.

#### Benton Facial Recognition Test

The Benton Facial Recognition Test requires simultaneous matching of black and white pictures of unfamiliar faces.

#### Judgement of Emotion, Gender, Age, and Gaze Direction

For the judgement of emotional expression task, participants were shown pictures of people displaying different emotional expressions (happiness, sadness, anger, and fear). The participant’s task was to name the emotion given in a list with four possible answers.

In judgement of gender task, faces of males and females were presented simultaneously on a screen. Participants had to orally indicate the location of the male face.

For the judgement of age task, pictures of people of different age were presented and participants had to arrange them according to their age (from young to old).

In the judgement of gaze direction task, two faces were presented simultaneously and the task was to indicate which face was looking at the observer. Head and gaze direction was systematically varied.

## Appendix B.

**Table B1.** Reaction Times for Each Subject, Group Means, and Standard Errors Separated by Stimulus Type, Type of Manipulation, and Trial Type (same/different).

	Same trials					
	Faces		Houses		Sugar bowls	
	Blurred	Scrambled	Blurred	Scrambled	Blurred	Scrambled
Participant	Control group					
H.J.	1178	1205	935	1156	861	1022
A.S.	3066	1966	1767	1597	1160	1297
L.G.	1197	1021	994	781	974	1071
S.H.	1417	1181	1083	1334	1023	1000
A.L.	1236	1164	974	1053	766	837
L.E.	1257	1111	1135	1360	869	1067
Group means	1558	1275	1148	1214	942	1049
*SE*	303	141	127	115	57	61
Participant	Experimental group (congenital prosopagnosics)					
G.H.	3732	1710	2068	1681	1252	1088
B.T.	1073	910	871	878	748	724
L.O.	1255	1431	1004	965	825	889
S.G.	1282	1688	1204	1655	686	737
X.G.	1115	1649	1143	1165	1019	1022
M.H.	3289	2401	1509	1909	1303	1486
Group means	1958	1275	1300	1376	973	991
*SE*	495	197	177	175	107	116
	Different trials					
	Faces		Houses		Sugar bowls	
	Blurred	Scrambled	Blurred	Scrambled	Blurred	Scrambled
Participant	Control Group					
H.J.	1178	1205	935	1156	861	1022
A.S.	3066	1966	1767	1597	1160	1297
L.G.	1197	1021	994	781	974	1071
S.H.	1417	1181	1083	1334	1023	1000
A.L.	1236	1164	974	1053	766	837
L.E.	1257	1111	1135	1360	869	1067
Group means	1558	1275	1148	1214	942	1049
*SE*	303	141	127	115	57	61
Participant	Experimental group (congenital prosopagnosics)					
G.H.	3732	1710	2068	1681	1252	1088
B.T.	1073	910	871	878	748	724
L.O.	1255	1431	1004	965	825	889
S.G.	1282	1688	1204	1655	686	737
X.G.	1115	1649	1143	1165	1019	1022
M.H.	3289	2401	1509	1909	1303	1486
Group means	1958	1275	1300	1376	973	991
*SE*	495	197	177	175	107	116

## References

[R1] Avidan G., Hasson U., Malach R., Behrmann M. (2005). Detailed exploration of face-related processing in congenital
						prosopagnosia: 2. Functional neuroimaging findings.. Journal of Cognitive Neuroscience.

[R2] Bartlett J. C., Searcy J., Abdi H., Petersen M. A., Rhodes G. (2003). What are the routes to face recognition?. Perception of faces, objects, and scenes.

[R3] Barton J. J., Cherkasova M., O’Connor M. (2001). Covert recognition in acquired and developmental
						prosopagnosia.. Neurology.

[R4] Barton J. J., Press D. Z., Keenan J. P., O’Connor M. (2002). Lesions of the fusiform face area impair perception of facial
						configuration in prosopagnosia.. Neurology.

[R5] Behrmann M., Avidan G. (2005). Congenital prosopagnosia: Face-blind from birth.. Trends in Cognitive Sciences.

[R6] Behrmann M., Avidan G., Gao F., Black S. (2007). Structural imaging reveals anatomical alterations in
						inferotemporal cortex in congenital prosopagnosia.. Cerebral Cortex.

[R7] Behrmann M., Avidan G., Marotta J. J., Kimchi R. (2005). Detailed exploration of face-related processing in congenital
						prosopagnosia: 1. Behavioral findings.. Journal of Cognitive Neuroscience.

[R8] Benton A. L., Sivan A. B., Hamsher K., Varney N. R., Spreen O. (1983). Contribution to neuropsychological assessment..

[R9] Bentin S., DeGutis J. M., D’Esposito M., Robertson L.C. (2007). Too many trees to see the forest: Performance, event-related
						potential, and functional magnetic resonance imaging manifestations of
						integrative congenital prosopagnosia.. Journal of Cognitive Neuroscience.

[R10] Bentin S., Deouell L. Y., Soroker N. (1999). Selective visual streaming in face recognition: Evidence from
						developmental prosopagnosia.. NeuroReport.

[R11] Biederman I. (1987). Recognition-by-components: A theory of human image
						understanding.. Psychological Review.

[R12] Bodamer J. (1947). Die Prosop-Agnosie.. Archiv für Psychiatrie und Nervenkrankheiten.

[R13] Bombari D., Mast F. W., Lobmaier J. S. (2009). Featural, configural, and holistic face-processing strategies
						evoke different scan patterns.. Perception.

[R14] Cabeza R., Kato T. (2000). Features are also important: Contributions of featural and
						configural processing to face recognition.. Psychological Science.

[R15] Carbon C. C., Gruter T., Weber J. E., Lueschow A. (2007). Faces as objects of non-expertise: Processing of thatcherised
						faces in congenital prosopagnosia.. Perception.

[R16] Collishaw S. M., Hole G. J. (2000). Featural and configurational processes in the recognition of
						faces of different familiarity.. Perception.

[R17] de Gelder B., Rouw R. (2000). Configural face processes in acquired and developmental
						prosopagnosia: Evidence for two separate face systems?. NeuroReport.

[R18] de Hahn E. H. (1999). A familiar factor in the development of face recognition
						deficits.. Journal of Clinical and Experimental Beuropsychology.

[R19] Degutis J. M., Bentin S., Robertson L. C., D’Esposito M. (2007). Functional plasticity in ventral temporal cortex following
						cognitive rehabilitation of a congenital prosopagnosic.. Journal of Cognitive Neuroscience.

[R20] Diamond R., Carey S. (1986). Why faces are and are not special: An effect of
						expertise.. Journal of Experimental Psychology, General.

[R21] Dobel C., Bolte J., Aicher M., Schweinberger S. R. (2007). Prosopagnosia without apparent cause: Overview and diagnosis of
						six cases.. Cortex.

[R22] Dobel C., Putsche C., Zwitserlood P., Junghofer M. (2008). Early left-hemispheric dysfunction of face processing in
						congenital prosopagnosia: An MEG study.. PLoS ONE.

[R23] Duchaine B. C. (2000). Developmental prosopagnosia with normal configural
						processing.. NeuroReport.

[R24] Duchaine B. C., Dingle K., Butterworth E., Nakayama K. (2004). Normal greeble learning in a severe case of developmental
						prosopagnosia.. Neuron.

[R25] Duchaine B. C., Nakayama K. (2004). Developmental prosopagnosia and the Benton Facial Recognition
						Test.. Neurology.

[R26] Duchaine B. C., Nakayama K. (2005). Dissociations of face and object recognition in developmental
						prosopagnosia.. Journal of Cognitive Neuroscience.

[R27] Duchaine B. C., Nakayama K. (2006). Developmental prosopagnosia: A window to content-specific face
						processing.. Current Opinions in Neurobiology.

[R28] Duchaine B. C., Nieminen-von Wendt T., New J., Kulomaki T. (2003). Dissociations of visual recognition in a developmental agnosic:
						Evidence for separate developmental processes.. Neurocase.

[R29] Duchaine B. C., Weidenfeld A. (2002). An evaluation of two commonly used tests of unfamiliar face
						recognition.. Neuropsychologia.

[R30] Farah M. J., Tanaka J. W., Drain H. M. (1995). What causes the face inversion effect?. Journal of Experimental Psychology, Human Perception and
						Performance.

[R31] Farah M. J., Wilson K. D., Drain M., Tanaka J. N. (1998). What is “special” about face
						perception?. Psychological Review.

[R32] Fast K., Fujiwara E., Markowitsch H. J. Famous faces test - Ein Verfahren zur Erfassung
						semantischer Altgedächtnisleistungen.. Göttingen: Hogrefe.

[R33] Gruter T., Gruter M., Bell V., Carbon C. C. (2009). Visual mental imagery in congenital
						prosopagnosia.. Neuroscience Letters.

[R34] Gruter T., Gruter M., Carbon C. C. (2008). Neural and genetic foundations of face recognition and
						prosopagnosia.. Journal of Neuropsychology.

[R35] Haig N. D. (1984). The effect of feature displacement on face
						recognition.. Perception.

[R36] Kennerknecht I., Grueter T., Welling B., Wentzek S., Horst J., Edwards S. (2006). First report of prevalence of non-syndromic hereditary
						prosopagnosia (HPA).. American Journal of Medical Genetics Part A.

[R37] Kress T., Daum I. (2003). Developmental prosopagnosia: A review.. Behavioral Neurology.

[R38] Lange J., de Lussanet M., Kuhlmann S., Zimmermann A., Lappe M., Zwitserlood P. (2009). Impairments of biological motion perception in congenital
						prosopagnosia.. PLoS ONE.

[R39] Leder H., Bruce V. (2000). When inverted faces are recognized: The role of configural
						information in face recognition.. Quarterly Journal of Experimental Psychology Section a-Human
						Experimental Psychology.

[R40] Leder H., Carbon C.-C. (2005). When context hinders! Context superiority versus
						learn-test-compatibilities in face recognition.. Quarterly Journal of Experimental Psychology, Section A: Human
						Experimental Psychology.

[R41] Leder H., Carbon C.-C. (2006). Face-specific configural processing of relational
						information.. British Journal of Psychology.

[R42] Lobmaier J. S., Klaver P., Loenneker T., Martin E., Mast F. W. (2008). Featural and configural face processing strategies: Evidence from
						a functional magnetic resonance imaging study.. NeuroReport.

[R43] Lobmaier J. S., Mast F. W. (2007). Perception of novel faces: The parts have it!. Perception.

[R44] Lobmaier J. S., Mast F. W. (2008). Face imagery is based on featural
						representations.. Experimental Psychology.

[R45] Macho S., Leder H. (1998). Your eyes only? A test of interactive influence in the processing
						of facial features.. Journal of Experimental Psychology: Human Perception and
						Performance.

[R46] Marr D. (1982). Vision..

[R47] Maurer D., Le Grand R., Mondloch C. J. (2002). The many faces of configural processing.. Trends in Cognitive Sciences.

[R48] Mondloch C. J., Geldart S., Maurer D., Le Grand R. (2003). Developmental changes in face processing skills.. Journal of Experimental Child Psychology.

[R49] Nunn J. A., Postma P., Pearson R. (2001). Developmental prosopagnosia: Should it be taken at face
						value?. Neurocase.

[R50] Rakover S. S. (2002). Featural vs. configurational information in faces: A conceptual
						and empirical analysis.. British Journal of Psychology.

[R51] Rossion B., Dricot L., Devolder A., Bodart J. M., Crommelinck M., De Gelder B. (2000). Hemispheric asymmetries for whole-based and part-based face
						processing in the human fusiform gyrus.. Journal of Cognitive Neuroscience.

[R52] Schwaninger A., Lobmaier J. S., Collishaw S. M. (2002). Role of featural and configural information in familiar and
						unfamiliar face recognition.. Lecture Notes in Computer Sciences.

[R53] Schwarzer G., Huber S., Gruter M., Gruter T., Gross C., Hipfel M. (2007). Gaze behaviour in hereditary prosopagnosia.. Psychological Research.

[R54] Searcy J. H., Bartlett J. C. (1996). Inversion and processing of component and spatial-relation
						information of faces.. Journal of Experimental Psychology: Human Perception and
						Performance.

[R55] Sergent J. (1984). Configural processing of faces in the left and the right cerebral
						hemispheres.. Journal of Experimental Psychology: Human Perception and
						Performance.

[R56] Tanaka J. W., Farah M. J. (1993). Parts and wholes in face recognition.. Quarterly Journal of Experimental Psychology Section A: Human
						Experimental Psychology.

[R57] Tanaka J. W., Sengco J. A. (1997). Features and their configuration in face
						recognition.. Memory and Cognition.

[R58] Thomas C., Avidan G., Humphreys K., Jung K. J., Gao F., Behrmann M. (2009). Reduced structural connectivity in ventral visual cortex in
						congenital prosopagnosia.. Nature Neuroscience.

[R59] Warrington E. K., James M. (1992). Testbatterie für visuelle Objekt- und
						Raumwahrnehmung..

